# Enzyme-Instructed Self-Assembling Peptide Hydrogels for Sustained Subconjunctival Delivery of Dexamethasone: A Stereochemical Comparison of L- and D-Enantiomeric Peptides for Postoperative Ocular Anti-Inflammatory Therapy

**DOI:** 10.3390/gels12090850

**Published:** 2026-09-18

**Authors:** Shubhamkumar M. Baviskar, Deepakkumar Mishra, Lalitkumar Vora, Garry Laverty, Sreekanth Pentlavalli, Raghu Raj Singh Thakur

**Affiliations:** 1 School of Pharmacy, Queen’s University Belfast, Belfast BT9 7BL, UK; s.baviskar@qub.ac.uk (S.M.B.); dmishra01@qub.ac.uk (D.M.); l.vora@qub.ac.uk (L.V.); garry.laverty@qub.ac.uk (G.L.); 2 Centre for Pharmaceutical Engineering Science (CPES), School of Pharmacy, University of Bradford, Bradford BD7 1DP, UK

**Keywords:** enzyme-instructed self-assembly, dexamethasone, ocular drug delivery, D-amino acid peptide, hydrogel, sustained release, alkaline phosphatase, postoperative inflammation

## Abstract

Sustained postoperative anti-inflammatory therapy following intraocular surgery remains an unmet clinical need, as conventional topical corticosteroid eye drops suffer from poor bioavailability, patient non-compliance, and an inability to maintain therapeutic drug levels over the multi-week inflammatory resolution period. Here we report the development and characterization of alkaline phosphatase (ALP)-responsive enzyme-instructed self-assembling (EISA) peptide hydrogels based on the ultrashort amphiphilic sequence NapFFKY(p)-OH, covalently conjugated with dexamethasone (DEX) via an ester-cleavable succinyl linker, in both L- and D-enantiomeric forms, as injectable platforms for subconjunctival drug delivery. Both conjugates, L-NapFFK(DEX)Y(p)-OH and D-NapFFK(DEX)Y(p)-OH, were synthesized with chemical purity (87.3% and 89.8%, respectively) and theoretical DEX loading of 29.58% *w*/*w*. ALP-triggered gelation was confirmed for both enantiomeric gels at 2% *w*/*v*, with rheological characterization of the platform establishing a storage modulus plateau of approximately 294 Pa, a G′/G″ ratio of ~10:1, and gel failure at approximately 578% shear strain. Transmission electron microscopy revealed stereochemistry-dependent differences in nanofiber network architecture, with the D-enantiomer forming a comparatively looser, more porous fibrillar matrix than its L-counterpart. In vitro DEX release over 28 days demonstrated that covalent conjugation eliminates the burst release associated with physical drug entrapment (41.1% for the L physical mixture versus 14.2% and 8.3% for L- and D-conjugated systems, respectively, at 2% *w*/*v*), while D-amino acid stereochemistry provides an additional level of sustained release restraint. Proteinase K stability studies confirmed the exceptional proteolytic resistance of the D-enantiomer, retaining 92.6 ± 2.2% of intact peptide at 6 h compared with 25.3 ± 0.8% for the L-enantiomer. Ex vivo scleral permeation studies demonstrated a trend towards greater cumulative DEX permeation (10.6 vs. 6.7 µg) and tissue retention (5.6 vs. 3.2 µg) for the D vs. L-formulation over 24 h. Both formulations exhibited injection forces within acceptable limits for a 30-gauge needle for ophthalmic administration, with the D-enantiomer requiring lower force than its L-counterpart. Cell viability of Dex-conjugated peptide sequence demonstrated non toxic in ARPE-19 human retinal pigment epithelial cells across all conditions tested, confirming biocompatibility. These findings establish the backbone stereochemistry independently controls network mechanics, proteolytic stability, and drug release rate in ALP-responsive EISA hydrogels. Cumulative release over 28 days remains below that required to cover a 4–6-week postoperative course, and this work is therefore presented as a mechanistic proof-of-concept defining design principles for the platform rather than a clinically optimized formulation. Drug loading, peptide sequence, and linker chemistry are identified as the levers through which release rate may be matched to intended clinical interval.

## 1. Introduction

Cataract extraction with intraocular lens (IOL) implantation is the most frequently performed elective surgical procedure worldwide, with an estimated 10 million operations performed annually [1]. Despite its high success rate, the surgical trauma inherent to phacoemulsification triggers a reproducible inflammatory cascade within the anterior chamber, characterized by disruption of the blood-aqueous barrier, release of prostaglandins and cytokines, and influx of inflammatory cells into the aqueous humor [2]. If inadequately controlled, this postoperative inflammation can progress to cystoid macular edema, posterior capsule opacification, and prolonged visual rehabilitation, all of which represent significant clinical and economic burdens [3]. Beyond routine cataract surgery, analogous inflammatory sequelae are encountered following refractive lens exchange, corneal transplantation, and trabeculectomy, underscoring the broad clinical need for robust, long-acting anti-inflammatory strategies in anterior segment care [2,4].

The current standard of care for postoperative anterior segment inflammation relies on topical corticosteroid eye drops, most commonly dexamethasone (DEX) or prednisolone acetate, administered multiple times daily for four to six weeks [5,6]. This regimen, while pharmacologically effective, is beset by well-documented limitations. The precorneal drainage, nasolacrimal clearance, and dilution by tear turnover collectively restrict topical bioavailability to less than 5% of the instilled dose [7]. Patients, particularly the elderly who constitute the predominant cataract-operated population, frequently struggle with accurate self-administration, resulting in subtherapeutic dosing and inconsistent anterior chamber drug levels [8]. Furthermore, preservatives such as benzalkonium chloride cause iatrogenic dry eye disease and corneal staining that complicates postoperative assessment [9]. These cumulative shortcomings have driven growing clinical interest in dropless or reduced-drop perioperative strategies that deliver drug directly and durably to the anterior chamber [10].

Two principal dropless strategies have been explored to address these limitations: intracameral and periocular (subconjunctival) drug delivery. Intracameral administration, in which a therapeutic agent is deposited directly into the anterior chamber at the time of surgery, bypasses ocular surface barriers entirely and achieves immediate, high local drug concentrations [11]. Intracameral DEX has been shown to produce intraocular drug concentrations orders of magnitude higher than topical administration, with several sustained-release intracameral platforms developed to capitalize on this advantage, including Surodex (Oculex Pharmaceuticals), a biodegradable PLGA rod implant sustaining DEX delivery for up to 10 days, and more recent thermoresponsive in situ-gelling systems offering multi-drug release over 30 to 40 days [12]. However, intracameral delivery carries risks specific to direct anterior chamber placement, including toxic anterior segment syndrome, endophthalmitis, and intraocular pressure spikes associated with retained implant material, and requires administration under sterile intraoperative conditions [13,14]. Subconjunctival injection, by contrast, offers a periocular depot route with a well-established clinical safety profile, exemplified by widespread use of subconjunctival triamcinolone and other corticosteroid depots, and can be administered as a straightforward office-based procedure either intraoperatively or postoperatively, without the intraocular complication risk profile of intracameral placement [10,15]. Given this more favorable risk–benefit balance and ease of clinical translation, the present study targets the subconjunctival route as the primary clinical application for this platform.

Enzyme-instructed self-assembly (EISA) has emerged as a powerful strategy for constructing supramolecular hydrogels directly within biological environments [16,17]. In the EISA paradigm, a soluble, non-assembling peptide precursor undergoes enzymatic transformation, typically dephosphorylation, to generate a self-assembling hydrogelator that spontaneously organizes into a three-dimensional nanofiber network under physiological conditions [18,19,20]. This approach is inherently bioorthogonal, exploits endogenous enzymes overexpressed at disease sites, and avoids the harsh crosslinking chemistry associated with conventional hydrogels, making it particularly attractive for sensitive ocular applications [21]. Alkaline phosphatase (ALP) is a naturally occurring phosphomonoesterase, with elevated activity broadly associated with ocular inflammation and surgical trauma [22,23,24]. ALP-responsive EISA has previously been demonstrated in the ocular context by Hu and colleagues, who showed that a phosphorylated peptide-ibuprofen conjugate undergoes ALP-triggered dephosphorylation in tear fluid to form a supramolecular hydrogel sustaining anti-inflammatory drug release over 96 h [25]. In the present study, ALP was supplied exogenously at supraphysiological activity to trigger assembly under controlled in vitro conditions; the concentrations used are not intended to represent physiological enzyme availability, and this is discussed further in Section 4.6 and as a limitation in 2.8. A critical but underexplored dimension of peptide hydrogelator design for in vivo applications is stereochemical identity. Natural L-amino acid peptides are inherently susceptible to proteolytic degradation by the broad-spectrum proteases present in ocular tissues and fluids, which limits the duration of drug release and gel persistence in situ [26]. The synthesis of peptide sequences composed entirely of D-amino acids confers exceptional resistance to proteolytic cleavage by virtue of the inverted stereochemistry, which is not recognized by most endogenous proteases [24]. Landmark studies by the Xu group demonstrated that D-amino acid incorporation into self-assembling peptide hydrogelators substantially prolongs in vitro and in vivo biostability while preserving gelation behavior and biocompatibility [27,28]. More recently, systematic D-amino acid substitution studies have confirmed that increasing D-residue content in peptide crosslinkers progressively extends hydrogel lifetime under enzymatic challenge [29,30]. Despite this body of evidence, no study has directly applied D-amino acid EISA hydrogels to periocular anterior segment drug delivery, nor systematically compared the stereochemistry-dependent release, stability, and ocular biocompatibility of DEX-conjugated L- and D-peptide hydrogels in a unified experimental framework. This D-amino acid design strategy builds on our group’s prior development of ALP- and phosphatase-responsive D-peptide hydrogel platforms for sustained systemic drug delivery [22,23,31], which established the proteolytic stability and controlled-release advantages of this scaffold class outside the ocular setting.

The present study addresses this gap by designing, synthesizing, and characterizing a library of ALP-responsive EISA hydrogels based on the ultrashort amphiphilic peptide sequence NapFFKY(p)-OH in both L- and D-enantiomeric forms, with DEX covalently conjugated to the lysine residue via an ester-cleavable succinyl linker. Herein, we report the synthesis, physicochemical characterization, and in vitro and ex vivo evaluation of L- and D-enantiomeric NapFFKY(p)-OH hydrogels covalently conjugated with DEX, with a view to establishing the impact of peptide stereochemistry on release kinetics, proteolytic stability, and ocular biocompatibility. This work presents, to our knowledge, the first direct comparison of L- and D-amino acid ALP-responsive EISA hydrogels as injectable platforms for subconjunctival sustained DEX delivery in the context of postoperative anterior segment inflammation [27,29].

## 2. Results and Discussion

### 2.1. Peptide Synthesis, DEX Conjugation, and Chemical Characterization

**Figure 1:**
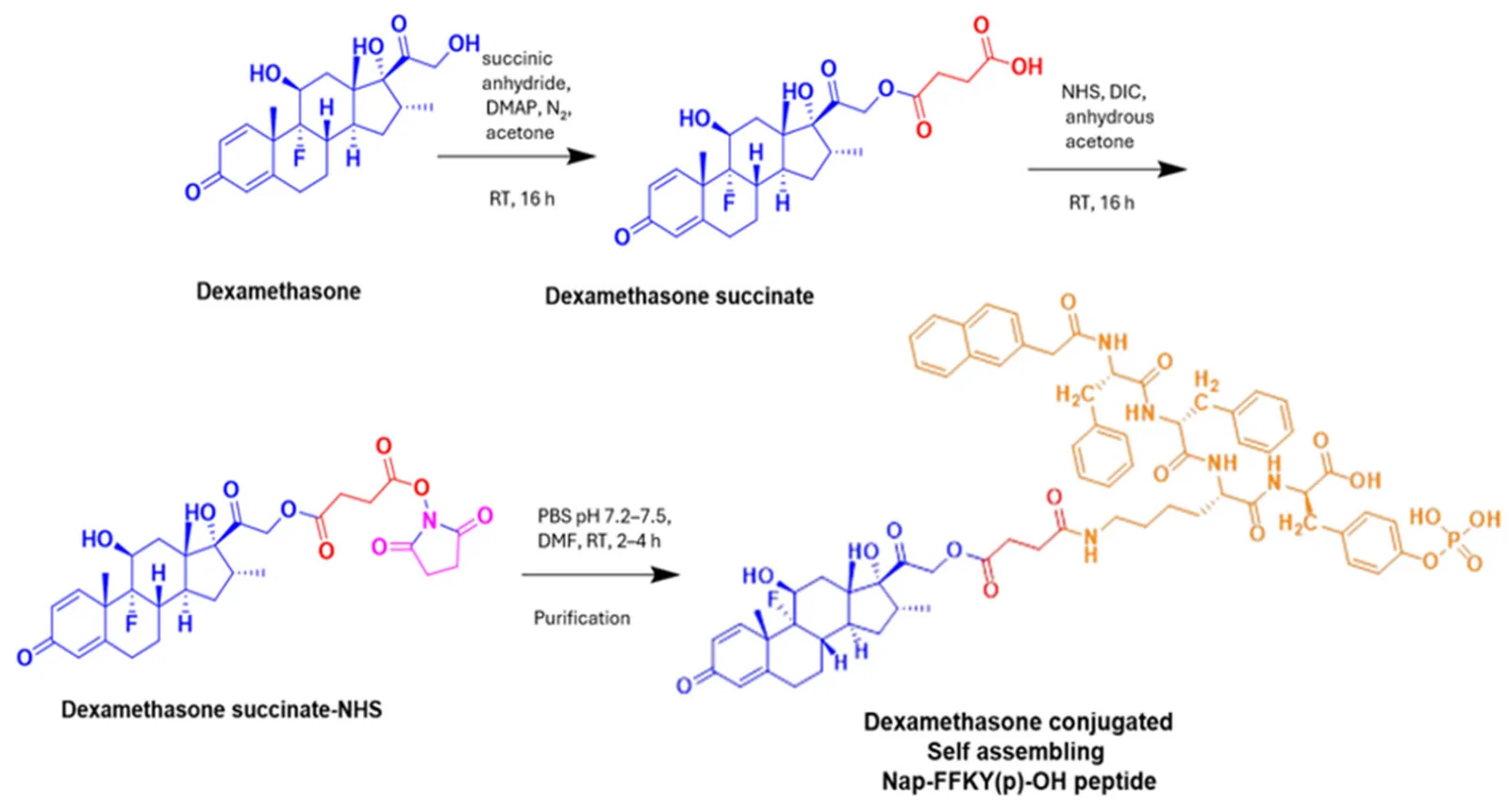
Synthesis and characterization of L- and D-NapFFK(DEX)Y(p)-OH conjugates. Synthetic route showing: esterification of DEX with succinic anhydride (DMAP, acetone, RT, 16 h) to yield DEX-21-succinate; NHS activation (NHS, DIC, acetone, RT, 16 h) to afford DEX-succinate-NHS; conjugation of the activated DEX ester to the ε-amino group of the lysine residue of L- or D-NapFFKY(p)-OH (PBS pH 7.2–7.5, RT, 2–4 h), followed by preparative RP-HPLC purification and vacuum drying.

L-NapFFKY(p)-OH and D-NapFFKY(p)-OH were successfully synthesized by Fmoc SPPS and confirmed by ESI-MS and RP-HPLC prior to conjugation (Figure 1). The observed *m*/*z* values for the parent sequences were in close agreement with theoretical masses (mass difference < 0.1 Da), and RP-HPLC confirmed purities of 99.3% and 100% for the L- and D-enantiomers, respectively, indicating high synthetic fidelity. The sequential chemical modification of DEX proceeded cleanly: esterification with succinic anhydride yielded DEX-21-succinate in approximately 95% yield, and subsequent NHS activation afforded DEX-succinate-NHS, confirmed by ESI-MS (observed *m*/*z* 591.24 [M+H]^+^; calculated 590.23). Conjugation of the activated DEX ester to the ε-amino group of the lysine residue in both L- and D-peptide sequences was confirmed by ESI-MS, with an observed *m*/*z* of 1326.54 [M+H]^+^ for both conjugates against a theoretical mass of 1325.54 Da. RP-HPLC purity of the final conjugates was 87.3% for L-NapFFK(DEX)Y(p)-OH and 89.8% for D-NapFFK(DEX)Y(p)-OH, with a theoretical DEX loading of 29.58% *w*/*w* (Figure 1, Table 1). This purity falls below the ≥95% threshold typically targeted for biomaterial-grade peptide conjugates; residual impurities cannot be excluded as a contributing factor to the release and toxicity data reported below, and improved purification is planned for future syntheses.

Structural confirmation was provided by ^1^H and ^31^P NMR spectroscopy. The ^1^H NMR spectrum of D-NapFFK(DEX)Y(p)-OH exhibited characteristic aromatic resonances in the region δ 7.0–9.2 ppm attributable to the phenylalanine benzyl protons, tyrosine aromatic protons, and the naphthalene moiety, with the complex multiplet integrating to approximately 31 protons consistent with the expected structure. Phosphorylation of the tyrosine residue was confirmed by ^31^P NMR, which showed a single phosphate resonance in both enantiomeric sequences, confirming successful and complete phosphorylation at the tyrosine hydroxyl, a prerequisite for ALP-responsive EISA behavior [25]. Together, these data confirm the synthesis of structurally defined, phase-pure L- and D-enantiomeric DEX-conjugated hydrogelator precursors amenable to enzyme-triggered self-assembly (Figure 1, Table 1).

### 2.2. ALP-Triggered Gelation, Nanostructure, and Mechanical Characterization of the EISA Platform

**Figure 2:**
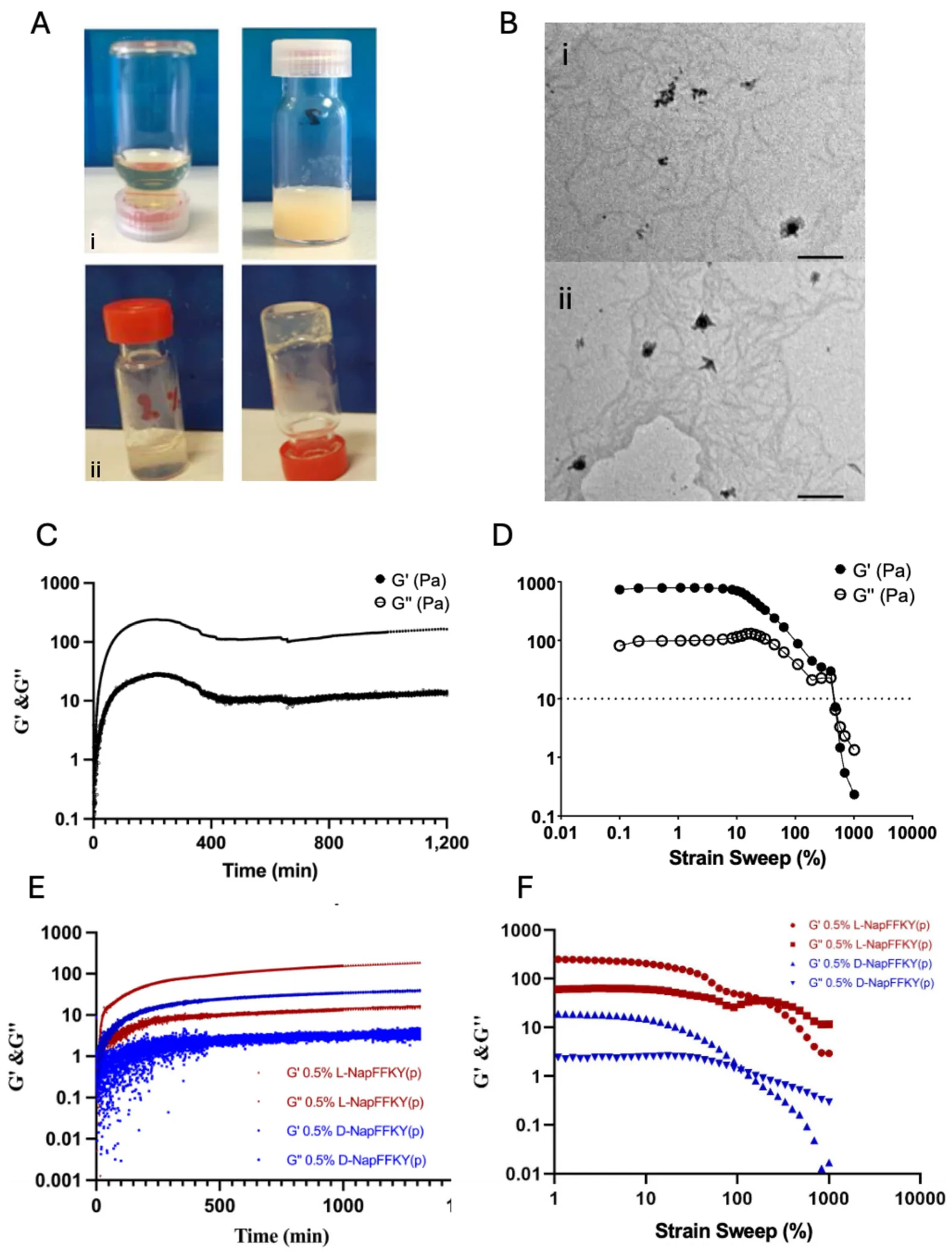
Hydrogel formation, nanostructure, and mechanical characterization of the EISA platform. (**A**) Invert vial gelation assay confirming gel formation for (**i**) L-NapFFK(DEX)Y(p)-OH and (**ii**) D-NapFFK(DEX)Y(p)-OH at 2% *w*/*v* following ALP addition (10 U, 37 °C, 24 h). (**B**) TEM images of (**i**) L- and (**ii**) D-enantiomeric hydrogels at 2% *w*/*v* showing stereochemistry-dependent differences in fibrillar network architecture; scale bar = 200 nm. (**C**) Oscillatory time sweep showing sol–gel transition for L-NapFFK(DEX)Y(p)-OH at 2% *w*/*v* following ALP addition; G′ (filled circles) and G″ (open circles) are shown. (**D**) Strain sweep confirming the linear viscoelastic region (LVR) and gel failure point. (**E**) Oscillatory time sweep comparing the unconjugated parent sequences L-NapFFKY(p)-OH and D-NapFFKY(p)-OH at 0.5% *w*/*v* following ALP addition, showing G′ and G″ for each enantiomer. (**F**) Strain sweep for both parent sequences at 0.5% *w*/*v*, showing the linear viscoelastic region and network failure point. The D-enantiomer forms the mechanically weaker network in both measurements, consistent with the looser fibrillar architecture seen by TEM. Rheology conducted on the L-enantiomer as the representative formulation; both enantiomers formed self-supporting gels by invert vial assay, though TEM (panel B) reveals a comparatively looser, more porous nanofiber network for the D-enantiomer. Comparative D-enantiomer rheology was not performed in the present study for conjugated sequence.

The capacity of both L-NapFFK(DEX)Y(p)-OH and D-NapFFK(DEX)Y(p)-OH to undergo ALP-triggered gelation was confirmed by the invert vial assay at 2% *w*/*v*. Both enantiomers formed self-supporting, cohesive hydrogels within 24 h of ALP addition at 37 °C, with neither formulation exhibiting flow upon inversion. This observation is consistent with the well-established EISA mechanism for NapFFKY(p)-based sequences, wherein enzymatic dephosphorylation of the phosphotyrosine residue renders the peptide more hydrophobic, driving intermolecular assembly into a supramolecular hydrogel network [18,23,29]. The minimum gelation concentration (MGC), defined as the concentration at which formulations reliably passed the full inversion test across replicates, was established at 2% *w*/*v* (Figure 2A) which was firm soft gel and this concentration was therefore selected as the working concentration for subsequent characterisation. This study also evaluated 0.5% *w*/*v* formulation which formed a soft gel which had lower mechanical strength, this lower concetration was additionally investigated in the study. Gel onset, evidenced by measurable viscosity increase and partial network formation, was observed at concentrations as low as 0.5% *w*/*v*, though these formulations did not reliably pass the strict inversion criterion. Sub-MGC self-assembly has previously been reported for structurally related Nap-Phe-Phe-pTyr-based hydrogelators, where nanostructure formation was detected by TEM and static light scattering at concentrations substantially below the formally defined MGC [32]. This lower-concentration self-assembly regime is exploited in Section 2.3, where 0.5% *w*/*v* was selected for comparative release studies.

TEM imaging of the formed hydrogels at 2% *w*/*v* revealed distinct morphological differences between the two enantiomers (Figure 2B). L-NapFFK(DEX)Y(p)-OH assembled into a dense, uniformly distributed fibrillar network with tightly interwoven nanofibers, indicative of strong and cooperative intermolecular interactions, consistent with the well-established propensity of L-amino acid aromatic peptides to form ordered, high-density self-assembled architectures (Figure 2B) [33]. In contrast, D-NapFFK(DEX)Y(p)-OH produced a comparatively looser, more porous fibrillar network with greater inter-fiber spacing (Figure 2(Bii)). This stereochemistry-dependent difference in network architecture is in agreement with previous reports demonstrating that D-amino acid enantiomers of self-assembling peptides can adopt subtly altered packing geometries due to their inverted chirality, resulting in less densely crosslinked supramolecular networks [31,34].

Oscillatory rheology of the DEX-conjugated system was performed on L-NapFFK(DEX)Y(p)-OH at 2% *w*/*v* as the representative formulation to characterize the viscoelastic properties of the drug-loaded hydrogel sequence. The time sweep confirmed a rapid sol–gel transition immediately following ALP addition, with G′ exceeding G″ within the first measurement interval. The storage modulus increased progressively, reaching a stable plateau of approximately 294 Pa (G′/G″ ratio ~10:1) at 63.1 min, indicating full network maturation. Frequency sweep analysis demonstrated that G′ remained consistently greater than G″ across the full range of angular frequencies tested (1–100 rad/s), with G′ values between 608 and 921 Pa, confirming frequency-independent elastic behavior characteristic of a true hydrogel [35]. Strain sweep analysis revealed a linear viscoelastic region up to approximately 10% strain, beyond which the network began to yield, with gel failure (G″ > G′) occurring at approximately 578% strain. This high tolerance to shear deformation before irreversible breakdown is advantageous for an injectable formulation, which must survive the mechanical stresses of passage through a fine-gauge needle while reforming upon deposition [30]. To establish whether the stereochemistry-dependent difference in network architecture observed by TEM corresponds to a measurable difference in bulk mechanical properties, oscillatory rheology was performed on the unconjugated parent sequences L-NapFFKY(p)-OH and D-NapFFKY(p)-OH at 0.5% *w*/*v* under identical ALP-triggered conditions (Figure 2 E,F). Both sequences formed viscoelastic networks with G′ exceeding G″ by approximately an order of magnitude throughout, confirming gel character. The two enantiomers were not, however, mechanically equivalent: L-NapFFKY(pP)-OH reached a plateau storage modulus of 197.1 Pa, approximately fivefold higher than the 38.7 Pa recorded for D-NapFFKY(p)-OH, and tolerated greater deformation before hydrogel network breakdown. The D-enantiomer therefore forms the mechanically weaker and less strain-tolerant network, in direct agreement with the looser, more porous fibrillar architecture observed by TEM (Figure 2B) and with lower injection force required for the D-conjugate through a 30-gauge needle (Section 2.6). As rheological characterization was performed only for the drug-conjugated L-enantiomer, this mechanical profile and the associated injectability inference should not yet be assumed to extend to the D-enantiomer, given its distinct network architecture; comparative D-enantiomer rheology, including shear recovery/thixotropy testing, is identified as a priority for future work

### 2.3. In Vitro Drug Release: Effect of Conjugation Strategy, Stereochemistry, and Concentration

**Figure 3:**
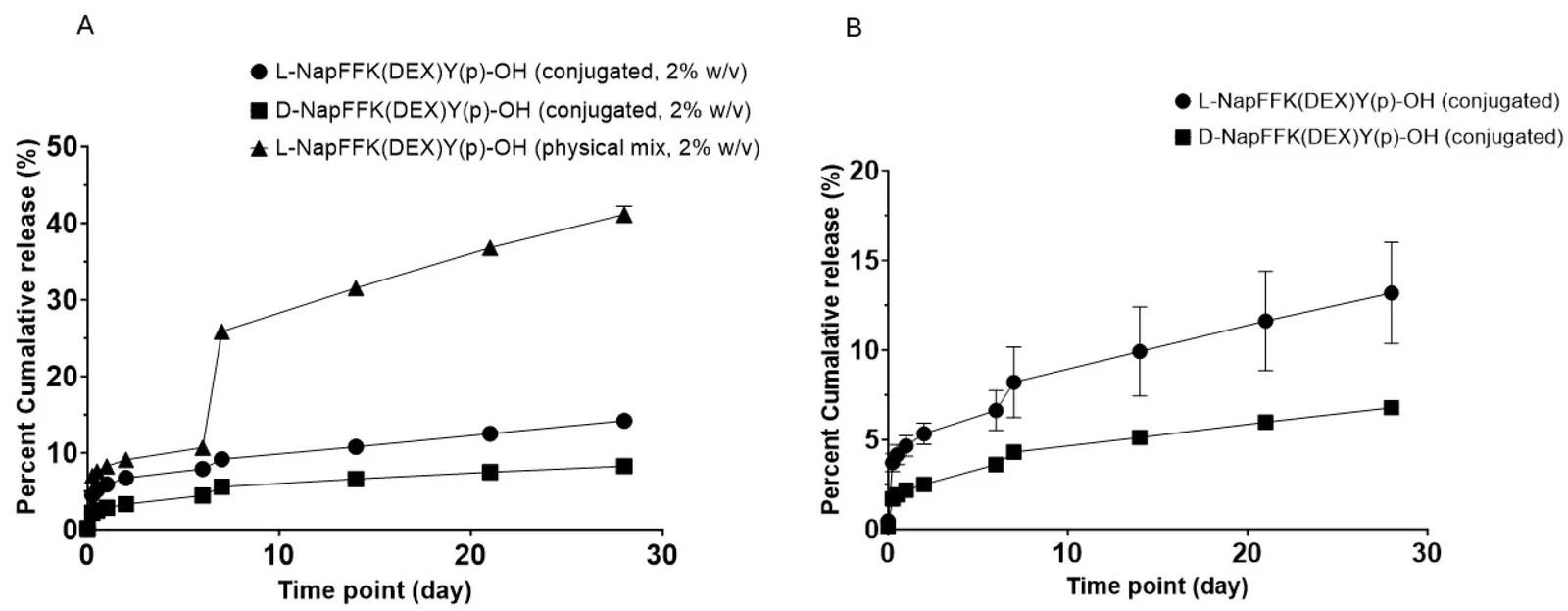
In vitro DEX release from L- and D-enantiomeric EISA hydrogels over 28 days under sink conditions (PBS, pH 7.4, 37 °C, 40 rpm). (**A**) Cumulative % DEX release at 2% *w*/*v* for L-NapFFK(DEX)Y(p)-OH physical mixture, L-NapFFK(DEX)Y(p)-OH chemically conjugated, and D-NapFFK(DEX)Y(p)-OH chemically conjugated. (**B**) Cumulative % DEX release at 0.5% *w*/*v* for L- and D-conjugated formulations. Data represent mean ± SD, *n* = 3.

In vitro DEX release was evaluated over 28 days under sink conditions (Figure 3A,B) to distinguish between the contributions of covalent conjugation, peptide stereochemistry, and formulation concentration to release kinetics. Three formulations were evaluated at 2% *w*/*v*: L-NapFFK(DEX)Y(p)-OH physical mixture, L-NapFFK(DEX)Y(p)-OH chemically conjugated, and D-NapFFK(DEX)Y(p)-OH chemically conjugated. The L physical mixture exhibited a rapid initial release, reaching approximately 10.7% by day 6, followed by a pronounced burst between days 6 and 7 (rising to 25.9%), and continuing to 41.1% cumulative release by day 28 (Figure 3A). This biphasic pattern is indicative of an initial diffusion-controlled phase followed by hydrogel network erosion, at which point physically entrapped drug is released rapidly as the gel matrix loses structural integrity. In contrast, the chemically conjugated L-formulation demonstrated markedly more controlled release, reaching only 14.2% over the same 28-day period, while the D-conjugated formulation released 8.3%, the lowest of all three systems. These data demonstrate two independent mechanisms driving sustained release: first, covalent attachment of DEX via the ester-cleavable succinyl linker eliminates the burst release associated with physical entrapment; second, D-amino acid stereochemistry provides an additional level of restraint on release rate. Notably, this restraint occurs despite the D-enantiomer forming a comparatively looser, more porous nanofiber network than the L-enantiomer (Section 2.2, Figure 2B), indicating that the slower D-form release cannot be attributed to reduced network porosity or a diffusion-limiting pathway. Instead, this is consistent with reduced susceptibility of the D-peptide backbone to hydrolytic and proteolytic cleavage, directly supported by the proteinase K biostability data in Section 2.4.

Cumulative release from both conjugated formulations remained insufficient at 28 days, and the release rate achieved here is therefore lower than that required to sustain anti-inflammatory action across a 4–6-week postoperative course. The present formulations are accordingly not proposed as clinically deployable dosage forms. The mechanistic findings above nonetheless identify the peptide–drug conjugate, rather than network density or peptide concentration, as the parameter that must be engineered to achieve therapeutic release rate.

To assess whether release behaviour depends on network density (concentration dependent), the chemically conjugated formulations were also evaluated at 0.5% *w*/*v* (Figure 3B). The L-conjugated gel released 13.2% and the D-conjugated gel 6.8% over 28 days, release profiles closely parallel to 2% *w*/*v* but similar cumulative values at equivalent timepoints, consistent with the reduced network density of gels at lower concentration. The D-enantiomer retained its advantage over the L-enantiomer at both concentrations, confirming that the stereochemical effect on release is intrinsic to the peptide–drug bond rather than an artifact of concentration-dependent network architecture. These findings align with recently reported D-amino acid peptide hydrogels for drug delivery, where D-peptides consistently exhibited slower and more linear release compared with their L-counterparts [27,28,31].

The effect of proteolytic activity on drug conjugated sequence was examined for L-NapFFK(DEX)Y(p)-OH at 0.5% *w*/*v* in the presence of proteinase K (PK, ~3 U/mL) added to the release medium. Under these conditions, cumulative DEX release reached approximately 29% over 28 days, substantially greater than the 13.2% observed in the absence of PK. This accelerated release in the presence of PK directly demonstrates that proteolytic degradation of the L-peptide backbone amplifies drug release by simultaneously accelerating hydrogel erosion and peptide–drug bond cleavage, underscoring the rationale for employing D-amino acid sequences that are inherently resistant to proteolytic cleavage [27]. Together with the network architecture data in Section 2.2, this indicates that DEX release from this platform is governed primarily by peptide backbone susceptibility to cleavage rather than by hydrogel pore size or diffusional pathway, explaining why the more porous D-enantiomer network nonetheless releases DEX more slowly than the denser L-network.

### 2.4. Proteolytic Stability of L- and D-Enantiomeric Sequences

**Figure 4:**
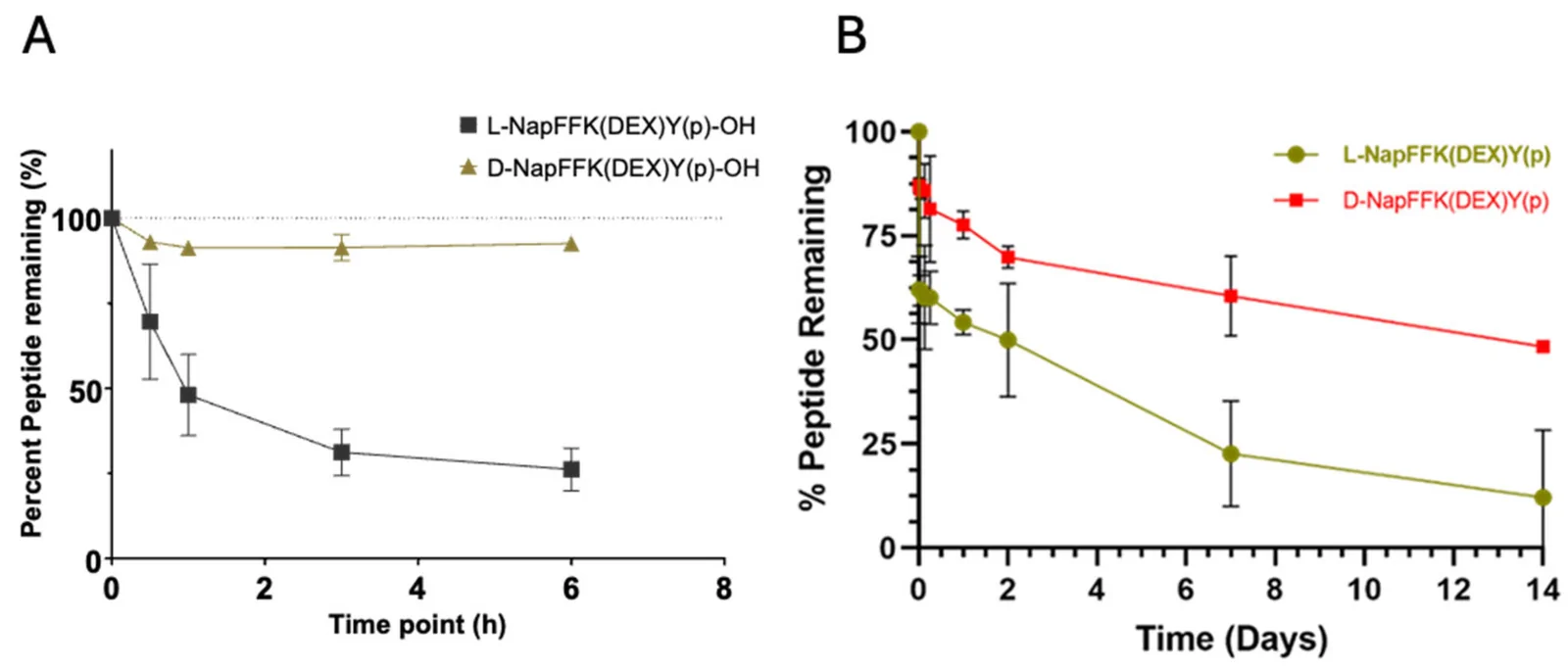
Proteolytic stability of L- and D-enantiomeric peptide sequences in the presence of proteinase K (PK, ~3 U/mL, 37 °C). (**A**) Percentage of intact L-NapFFKY (p)-OH and D-NapFFKY(p)-OH parent sequences remaining over 6 h, quantified by RP-HPLC peak area relative to t = 0. D-amino acid stereochemistry confers near-complete resistance to PK-mediated cleavage (92.6 ± 2.2% remaining at 6 h) compared with rapid degradation of the L-enantiomer (25.3 ± 0.8% at 6 h). (**B**) Longer-term stability of DEX-conjugated sequences over 14 days; D-NapFFK(DEX)Y(p)-OH retained approximately 48.37 ± 1.12% of its initial peak area versus 12.15 ± 16.21% for the L-conjugate. Dashed horizontal line at 100% represents no-enzyme control. Data represent mean ± SD, *n* = 3.

The susceptibility of the parent unloaded peptide sequences and their DEX conjugates to proteinase K-mediated degradation was assessed by RP-HPLC over an extended timeframe. For the parent sequences at 0.02% *w*/*v*, the L-enantiomer degraded rapidly, retaining only 67.5 ± 3.6% of its initial peak area by 0.5 h, 46.5 ± 1.4% at 1 h, 30.3 ± 0.5% at 3 h, and 25.3 ± 0.8% at 6 h. In stark contrast, D-NapFFKY(p)-OH demonstrated exceptional resistance throughout, retaining 93.0 ± 1.5% at 0.5 h, 91.3 ± 0.2% at 1 h, 91.3 ± 2.3% at 3 h, and 92.6 ± 2.2% at 6 h (Figure 4A), statistically indistinguishable from the t = 0 value, indicating that the D-enantiomer is essentially inert to PK-mediated cleavage under these conditions. This finding is in agreement with the seminal work of Li and colleagues [28] and with more recent systematic studies confirming that D-amino acid incorporation significantly extends peptide lifetime in enzymatic environments [30].

For the DEX-conjugated sequences, the longer-term study confirmed that L-NapFFK(DEX)Y(p)-OH was substantially degraded, retaining only 12.15 ± 16.21%of its initial structure by day 14 (Figure 4B). In contrast, D-NapFFK(DEX)Y(p)-OH remained 48.37 ± 1.12% at the same timepoint. This reduction in D-conjugate stability relative to the parent D-peptide suggests that the presence of the bulky DEX-succinate moiety may subtly alter nanofiber packing or expose the peptide backbone to limited enzymatic access, as has been noted in other drug-conjugated peptide systems [27]. Nevertheless, the D-conjugate retains a substantial stability advantage over its L-counterpart, directly relevant to the extended release performance observed above.

This finding is in agreement with the seminal work of Li and colleagues [36] and with more recent systematic studies confirming that D-amino acid incorporation significantly extends peptide lifetime in enzymatic environments [28]. As noted in Section 4.9, proteinase K is a broad-spectrum surrogate rather than an ocularly specific protease; the stability advantage demonstrated here should be interpreted as evidence of general proteolytic resistance, with confirmation against ocularly relevant proteases identified as a priority for future work.

### 2.5. Ex Vivo Scleral Permeation and Tissue Retention

**Figure 5:**
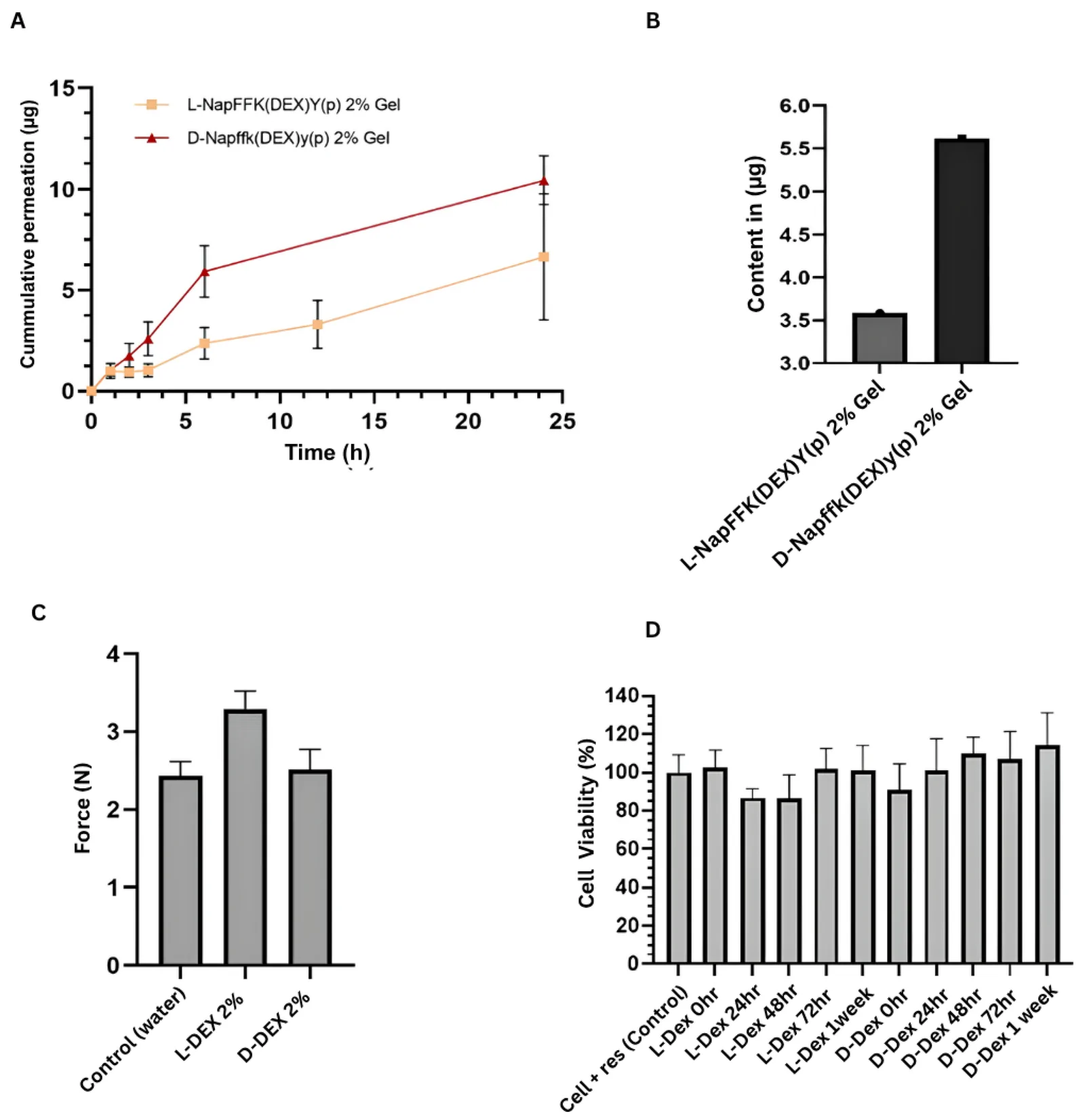
Translational characterization of L- and D-NapFFK(DEX)Y(p)-OH hydrogels at 2% *w*/*v*. (**A**) Cumulative DEX permeation (in µg) across porcine conjunctival–scleral tissue over 24 h in a modified Franz diffusion cell model (mean ± SD, *n* = 3; no significant difference between enantiomers, *p* > 0.05). (**B**) Scleral tissue retention of DEX at 24 h for L-NapFFK(DEX)Y(p)-OH and D-NapFFK(DEX)Y(p)-OH, showing significantly greater retention for the D-enantiomer (mean ± SD, *n* = 3) (**C**) Injection force-time profiles through a 30-gauge needle for water (control), L-NapFFK(DEX)Y(p)-OH 2% *w*/*v*, and D-NapFFK(DEX)Y(p)-OH 2% *w*/*v*; bar chart shows maximum injection force (N, mean ± SD, *n* = 3). (**D**) ARPE-19 human retinal pigment epithelial cell viability following indirect contact with formulation extracts at 0 h, 24 h, 48 h, 72 h and 1 week (L-NapFFKY(p)-OH, L-NapFFK(DEX)Y(p)-OH, D-NapFFKY(p)-OH, and D-NapFFK(DEX)Y(p)-OH), assessed by resazurin reduction assay and expressed as a percentage of untreated controls. Data represent mean ± SD, *n* = 3.

Ex vivo permeation and tissue retention of DEX from both conjugated hydrogel formulations were evaluated using excised porcine conjunctival–scleral tissue in a modified Franz diffusion cell system over 24 h. Both L- and D-NapFFK(DEX)Y(p)-OH hydrogels demonstrated time-dependent, sustained DEX permeation into the receptor compartment, consistent with gradual ester-bond hydrolysis and diffusion-controlled drug transport through the scleral membrane. D-NapFFK(DEX)Y(p)-OH demonstrated a trend towards greater cumulative permeation (10.6 ± 1.2 µg) compared with L-NapFFK(DEX)Y(p)-OH (6.7 ± 3.0 µg) over 24 h (Figure 5A), although this difference did not reach statistical significance at the final timepoint (*p* > 0.05). The observed trend is nonetheless consistent with the TEM observation of a more porous fibrillar network in the D-enantiomer gel, which may facilitate more efficient drug mobility within the depot and more sustained release at the tissue interface over longer timescales.

Quantification of DEX retained within the scleral tissue following the 24 h study revealed greater tissue accumulation for the D-enantiomer formulation: 3.6 µg for L-NapFFK(DEX)Y(p)-OH and 5.6 µg for D-NapFFK(DEX)Y(p)-OH (Figure 5B). Considered alongside the permeation data, the total DEX recovered across both compartments was 10.3 µg for the L-formulation and 16.2 µg for the D-formulation, suggesting that the D-conjugate hydrogel depot releases a greater cumulative quantity of drug over the 24 h study period. This is consistent with the superior proteolytic stability of the D-enantiomer scaffold demonstrated in Section 2.4. The D-gel maintains its structural integrity and continues releasing drug throughout the observation window, whereas the L-gel, being more susceptible to enzymatic degradation, may undergo earlier depot erosion, resulting in a smaller total drug output. These data collectively suggest that D-NapFFK(DEX)Y(p)-OH functions as a more durable and productive drug reservoir under biologically relevant conditions, a property highly desirable for a subconjunctival delivery system targeting the anterior segment [37].

### 2.6. Injectability

Both L- and D-NapFFK(DEX)Y(p)-OH formulations at 2% *w*/*v* were assessed for injectability through a 30-gauge needle (BD Emerald^TM^), internal diameter 0.159 mm, using a texture analyzer, with water as a baseline control for intrinsic plunger/syringe friction (Section 4.11). Maximum injection force was 3.29 N ± 0.23 N for L-NapFFK(DEX)Y(p)-OH and 2.51 N ± 0.25 N for D-NapFFK(DEX)Y(p)-OH (*p* = 0.0098) (Figure 5C). Both values are of the same order as those reported by Thakur et al. for in situ forming poloxamer implants administered via ocular routes [37] and fall well below the 20 N target recommended for manual parenteral injection [38], indicating that neither formulation presents a practical barrier to administration by hand. This mechanical differentiation is practically important: the reduced injection force of the D-formulation translates to improved ease of administration through fine-gauge needles, reducing patient discomfort and the risk of injection-related tissue damage in the sensitive intraocular environment.

### 2.7. In Vitro Biocompatibility with ARPE-19 Cells

Biocompatibility of all four peptide sequences was evaluated in ARPE-19 human retinal pigment epithelial cells by resazurin assay under both direct contact (24 h) and indirect conditions assessed at 0 h, 24 h, 48 h, 72 h and 1 week (Section 4.13), in accordance with ISO 10993-5:2009 [39]. Cell viability ranging from 86 ± 9% to 114 ± 15% exceeding 100% of untreated controls across all formulations and all timepoints assessed, with no statistically significant difference between L- and D enantiomeric DEX-conjugated forms. The maintenance of cell viability above control levels is consistent with reports of related self-assembling peptide matrices that provide a supportive microenvironment for anchorage-dependent cell types [7,40], confirming the biocompatibility of both enantiomeric platforms for ocular drug delivery applications based on viability as the primary endpoint; more sensitive markers of sublethal toxicity were not assessed in this study.

Taken together, the results establish D-NapFFK(DEX)Y(p)-OH as the superior candidate formulation across all key performance criteria: the most sustained DEX release profile under both standard and proteolytic conditions, the greatest scleral permeation and tissue retention, lower injection forces, and equivalent biocompatibility to the L-form (Figure 5C). These advantages are mechanistically underpinned by the proteolytic resistance conferred by D-amino acid stereochemistry, which preserves both the hydrogel depot structure and the peptide–drug ester bond over extended periods in enzymatic environments.

### 2.8. Limitations and Future Directions

While this study establishes proof-of-concept for L- and D-enantiomeric ALP-responsive peptide hydrogels as a platform for sustained subconjunctival delivery of dexamethasone, several limitations should be acknowledged, and corresponding priorities for future work identified. Comparative rheological characterization was performed only for the L-enantiomer in the present study, despite TEM data indicating a looser, more porous network for the D-enantiomer. Consequently, the relationship between network architecture and mechanical properties (storage modulus, yield strain, viscoelastic behavior) has not been directly established for the D-conjugate, and injectability and mechanical stability claims for this enantiomer should be interpreted with appropriate caution pending this comparison. Shear recovery (thixotropy) testing, which would further support injectability claims relevant to needle-based administration, was also not performed. Repeating full rheological characterization for both enantiomers under identical conditions, including replicate time- and frequency-sweep traces with reported mean ± SD, is identified as a priority for the next stage of this work. Alkaline phosphatase was supplied exogenously at supraphysiological activity to achieve rapid and complete in vitro gelation under controlled conditions. Enzyme concentration influences the kinetic pathway of self-assembly, and the architectures reported here should therefore be understood as those formed under rapid, complete dephosphorylation. Gelation driven by endogenous ALP at the subconjunctival site has not been demonstrated in this study, and quantitative ALP activity data for subconjunctival tissue are not available in the literature. We note, however, that the use of supraphysiological enzyme activity for invitro characterization does not preclude assembly under endogenous conditions: ALP-triggered self-assembly of a structurally related phosphopeptides–drug conjugate has been demonstrated in tear fluid [25], and the NapFFKY(p)OH scaffold employed here has been shown to form a functional hydrogel depot in vivo following subcutaneous administration, sustaining therapeutic plasma drug concentrations over an extended period [29]. Direct measurement of tissue ALP activity at the intended injection site, and demonstration of in situ gelation in vivo, are nonetheless required. The platform is also compatible with a two-component presentation in which enzyme and precursor are combined immediately before injection, removing dependence on endogenous enzyme availability.

In vitro release studies were performed in enzyme-free phosphate-buffered saline, which captures baseline chemical hydrolysis of the peptide–drug ester linkage but does not replicate esterase-mediated cleavage expected in vivo. The 28-day release data should therefore be interpreted as a measure of intrinsic hydrolytic behavior rather than a directly predictive in vivo release profile. Kinetic modeling of the release data (Korsmeyer–Peppas analysis) indicates that the L- and D-conjugates share a common release mechanism, with the slower release observed for the D-enantiomer attributable to a reduced rate constant rather than a distinct transport pathway, consistent with its superior proteolytic stability. However, this interpretation has not been directly confirmed at the molecular level; LC-MS monitoring to distinguish free dexamethasone from intact or partially hydrolyzed peptide–drug fragments in the release medium would provide direct mechanistic confirmation and is identified as a priority follow-up study. At the formulation concentration tested, cumulative drug release remained well below completion at 28 days, indicating that the release rate would need to be increased, through higher drug loading, peptide sequence modification, or alternative linker chemistry, to align with the clinically relevant 4–6-week postoperative anti-inflammatory window; the present data should accordingly be interpreted as establishing feasibility and mechanism rather than a clinically optimized release interval.

Cytotoxicity and biocompatibility were assessed using the ARPE-19 retinal epithelial cell line, which does not directly represent the anterior segment tissues (cornea, conjunctiva) relevant to subconjunctival administration; corneal epithelial cells or conjunctival fibroblasts would provide a more clinically representative model and are identified as a priority for future biocompatibility studies. Additionally, more sensitive endpoints, including markers of apoptosis and inflammatory cytokine release, alongside longer-term exposure, would strengthen the current viability-based toxicity assessment. Proteolytic stability was assessed using proteinase K, a broad-spectrum protease that is not fully representative of the ocular proteolytic environment; assessment against ocularly relevant proteases, or human aqueous humour/tear fluid protease mixtures, would allow in vivo biostability claims to be made with greater confidence. Ex vivo scleral permeation was assessed with a limited sample size (*n* = 3), and the resulting comparative trends between enantiomers should be regarded as preliminary; studies incorporating a larger number of biological replicates, ideally from multiple donor animals, alongside reporting of tissue thickness and donor variability, are needed to establish these differences with adequate statistical power.

Compound purity for both conjugates (87.3% L-form, 89.8% D-form) fell below the ≥95% threshold typically targeted for biomaterial-grade peptide conjugates in this platform, and residual impurities cannot be excluded as a contributing factor to the observed release and toxicity profiles; improved purification strategies are planned for future syntheses. Site-specific conjugation of dexamethasone to the peptide ε-amine was inferred from expected reaction selectivity rather than confirmed by MS/MS fragmentation analysis, and enzyme activity was assumed stable across the assay timeframe without direct verification; both are identified as straightforward additions for future structural and functional characterization. Similarly, injection force measurements did not include a blank plunger-friction baseline, meaning reported values reflect total force rather than the drug formulation’s contribution in isolation; inclusion of such a baseline would allow this effect to be normalized in subsequent studies.

Collectively, these limitations do not undermine the core finding of this study, that D-peptide stereochemistry confers measurable, mechanistically interpretable advantages in proteolytic stability, sustained release, and injectability relative to the L-enantiomer, but they define a clear set of priorities before this platform can progress toward in vivo validation: comparative D-enantiomer rheology and thixotropy testing, esterase-inclusive and mechanistically resolved release studies, anterior-segment-relevant biological models with expanded toxicity endpoints, ocularly relevant proteolytic stability testing, and completion of outstanding analytical characterization. Addressing these points, together with in vivo pharmacokinetic and efficacy evaluation in an appropriate ocular model, represents the next stage of development for this D-peptide hydrogel platform as a dropless, sustained-release postoperative anti-inflammatory therapy.

## 3. Conclusions

This study reports the design, synthesis, and systematic characterization of ALP-responsive enzyme-instructed self-assembling peptide hydrogels based on the NapFFKY(p)-OH scaffold, covalently conjugated with DEX via an ester-cleavable succinyl linker, in both L- and D-enantiomeric forms. Both conjugates were synthesized with high purity and confirmed molecular identity, and both underwent ALP-triggered gelation under physiological conditions, forming self-supporting nanofibrous hydrogel networks at 2% *w*/*v*. Rheological characterization confirmed that the EISA platform produces mechanically robust hydrogels with a storage modulus plateau of ~294 Pa and a wide linear viscoelastic region, with gel failure occurring only at approximately 578% strain, properties consistent with the demands of periocular injection and in situ depot formation.

The principal finding of this work is that backbone stereochemistry exerts a consistent and measurable influence across three independent classes of endpoints: network mechanics, 2. stability, and drug release rate. The most significant finding of this work is the consistent demonstration of the D-enantiomeric formulation across every performance criterion evaluated. D-NapFFK(DEX)Y(p)-OH demonstrated the lowest cumulative DEX release over 28 days (8.3% at 2% *w*/*v*; 6.8% at 0.5% *w*/*v*), the greatest resistance to proteinase K-mediated degradation (retaining 92.6% of intact peptide at 6 h versus 25.3% for the L-enantiomer), the highest scleral permeation (10.6 µg) and tissue retention (5.6 µg) in the ex vivo Franz cell model, and lower injection forces through a 30-gauge needle compared with the L-formulation. Biocompatibility was confirmed for both enantiomers in ARPE-19 retinal pigment epithelial cells, with cell viability ranging from 86 ± 9% to 114 ± 15% of untreated controls across both drug-conjugated sequences and timepoints (Figure 5D). These advantages collectively make D-NapFFK(DEX)Y(p)-OH a compelling candidate platform for sustained subconjunctival anti-inflammatory therapy following intraocular surgery.

The data further establish that covalent conjugation of DEX to the peptide hydrogelator via a hydrolyzable ester linkage is essential for controlled, sustained release. The L-enantiomer physical mixture exhibited a pronounced biphasic burst release reaching 41.1% over 28 days, with a sharp acceleration between days 6 and 7 attributable to hydrogel network erosion, in stark contrast to the monotonically sustained profiles of both chemically conjugated systems. This distinction is mechanistically informative: a burst-release formulation risks supra-therapeutic DEX concentrations in the early postoperative period while failing to provide coverage during later inflammatory resolution. In contrast, the conjugated D-peptide system offers a slow, controlled drug release profile, and the conjugated chemistry is identified as the principal determinant of release rate available for subsequent optimization. Such a release profile is therapeutically desirable for long-acting anterior segment anti-inflammatory therapy.

Several important directions remain to be addressed before clinical translation can be pursued. First, and most critically, in vivo pharmacokinetic and pharmacodynamic studies are required to validate the in vitro and ex vivo findings reported here. Subconjunctival administration of both L- and D-NapFFK(DEX)Y(p)-OH in a rabbit eye model, the accepted regulatory standard for ocular drug delivery studies, would allow quantification of aqueous humor DEX concentrations over time and direct correlation with anterior chamber inflammation endpoints, such as cell and flare scoring or prostaglandin E_2_ levels, following a standardized surgical insult [14]. Such studies would confirm whether the sustained release rates observed in vitro translate to therapeutically meaningful intraocular drug levels and anti-inflammatory efficacy in vivo.

Second, the in vivo gelation kinetics and gel persistence of the D-enantiomer formulation within the anterior chamber environment warrant direct investigation. While ALP activity has been documented in the aqueous humor of post-surgical eyes [24], the precise enzyme concentration at the injection site and its variability between patients will influence the rate and completeness of in situ hydrogelation. Anterior segment optical coherence tomography could be employed to monitor gel formation, spatial distribution, and gradual resorption non-invasively over time.

Third, the long-term biocompatibility and biodegradation profile of the peptide scaffold in vivo require characterization. The degradation products of the peptide backbone and DEX-succinate linker following hydrolysis should be assessed for ocular tolerability in vivo, including any potential effect on intraocular pressure, corneal endothelial cell density, and retinal integrity. Immunogenicity of the D-amino acid scaffold, while generally considered low owing to its resistance to antigen processing, should also be confirmed in an immune-competent model.

Finally, formulation optimization presents several opportunities to enhance performance further. The peptide concentration, DEX loading, and linker chemistry could be tuned to achieve different release durations matching the inflammatory time course of specific surgical procedures. Co-delivery of a complementary therapeutic agent, such as an antibiotic to address the concurrent infection risk in the postoperative period, within the same EISA scaffold represents a particularly attractive avenue, building on the demonstrated versatility of the NapFFKY(p) platform for multi-drug conjugation [29].

In summary, this work provides a preliminary comparative foundation for D-amino acid EISA hydrogels as a new class of stimuli-responsive, injectable drug delivery systems for sustained anterior segment therapy, and defines a clear translational roadmap for their further development towards clinical evaluation.

## 4. Materials and Methods

### 4.1. Materials

Wang resin (100–200 mesh, 1 mmol/g loading), O-(benzotriazol-1-yl)-N,N,N′,N′-tetramethyluronium hexafluorophosphate (HBTU), 1-hydroxybenzotriazole (HOBt), N-α-Fmoc-N-ε-Boc-L-lysine, N-α-Fmoc-N-ε-Boc-D-lysine, N-α-Fmoc-L-phenylalanine, N-α-Fmoc-D-phenylalanine, N-α-Fmoc-L-tyrosine, N-α-Fmoc-D-tyrosine, naphthalene-2-acetic acid (>98% purity), dichloromethane (DCM), N,N-dimethylformamide (DMF), N,N-diisopropylethylamine (DIPEA), piperidine, trifluoroacetic acid (TFA), triisopropylsilane (TIPS), thioanisole, diethyl ether, ninhydrin, pyridine, and deuterated dimethyl sulfoxide (DMSO-d6) were purchased from Merck/Sigma-Aldrich (Gillingham, UK). Dexamethasone (DEX) was purchased from Alfa Aesar (Heysham, UK). Succinic anhydride, N-hydroxysuccinimide (NHS), N,N′-diisopropylcarbodiimide (DIC), 4-dimethylaminopyridine (DMAP), anhydrous acetone, and alkaline phosphatase (ALP, bovine intestinal mucosa) were purchased from Sigma-Aldrich. Proteinase K from *Tritirachium album* (≥30 U/mg) was obtained from Sigma-Aldrich. Phosphate-buffered saline (PBS, pH 7.4) was prepared in-house. ARPE-19 human retinal pigment epithelial cells were obtained from the American Type Culture Collection (ATCC, Manassas, VA, USA). Dulbecco’s modified Eagle’s medium/nutrient mixture F-12 (DMEM/F-12), fetal bovine serum (FBS), and penicillin-streptomycin were purchased from Gibco (Thermo Fisher Scientific, Paisley, UK). Resazurin sodium salt was obtained from Sigma-Aldrich. All other solvents were of HPLC or analytical grade. Fresh porcine eyes were obtained from a local abattoir (Belfast, UK) on the day of experimentation.

### 4.2. Solid-Phase Peptide Synthesis of L- and D-NapFFKY(p)-OH

L-NapFFKY(p)-OH and D-NapFFKY(p)-OH peptide sequences were synthesized using standard Fmoc solid-phase peptide synthesis (SPPS) on Wang resin, following the protocol reported by Coulter et al. [29]. Briefly, Fmoc deprotection was carried out with 20% *v*/*v* piperidine in DMF (2 × 10 min). Coupling reactions employed HBTU/HOBt/DIPEA (4:4:8 equivalents relative to resin loading) in DMF for 60 min. Completion of each coupling step was confirmed by ninhydrin (Kaiser) test. Following assembly of the full sequence, the naphthalene-2-acetic acid capping group was coupled at the N-terminus under identical conditions. Phosphorylation of the tyrosine hydroxyl was achieved using phosphoric acid/pyridine in DMF. Cleavage from resin and side-chain deprotection were performed with a cocktail of 95% TFA, 2.5% TIPS, and 2.5% thioanisole for 3 h at room temperature. The crude peptide was precipitated in cold diethyl ether, collected by centrifugation, and lyophilized. The same protocol was applied using the corresponding D-amino acid building blocks to synthesize D-NapFFKY(p)-OH.

### 4.3. Synthesis of DEX-Succinate-NHS and Peptide–Drug Conjugation

DEX was chemically modified to introduce an ester-cleavable succinyl linker prior to conjugation, following the approach described by Vlieghe et al. [41] and adapted by Wang/Xu et al. [27]. Briefly, 1 g (2.55 mmol) of DEX, 1.2 g (11.99 mmol) of succinic anhydride, and 0.3 g (2.46 mmol) of DMAP were dissolved in anhydrous acetone under a nitrogen atmosphere and stirred overnight at room temperature. Acetone was removed under reduced pressure, and the residue was dissolved in ethanol/water (14:34 mL), stored at 4 °C for 48 h for crystallization, and the resulting DEX-21-succinate crystals were collected by vacuum filtration and dried (yield ~95%).

NHS esterification was performed by combining 1.2 g of DEX-succinate with 2 mmol NHS and 2 mmol DIC in anhydrous acetone and stirring overnight at room temperature. The reaction mixture was filtered and the filtrate concentrated under reduced pressure to yield DEX-succinate-NHS as white crystals. Successful synthesis was confirmed by ESI-MS (observed *m*/*z* 591.24 [M+H]^+^; calculated 590.23).

For peptide–drug conjugation, DEX-succinate-NHS was dissolved in DMF and added dropwise to the peptide (L- or D-NapFFKY(p)-OH) dissolved in PBS (pH 7.2–7.5), targeting reaction at the ε-amino group of the lysine residue. The mixture was stirred at room temperature for 2–4 h, and the resulting conjugates (L-NapFFK(DEX)Y(p)-OH and D-NapFFK(DEX)Y(p)-OH) were purified by preparative RP-HPLC and lyophilized.

### 4.4. Peptide Characterization by NMR, ESI-MS, and RP-HPLC

Structural confirmation of the synthesized peptides and DEX-conjugated sequences was performed by ^1^H NMR and ^31^P NMR spectroscopy (2 mg sample dissolved in DMSO-d6). ^1^H NMR confirmed the aromatic proton signals of the phenylalanine, tyrosine, and naphthalene moieties, and ^31^P NMR confirmed the presence of the phosphate group on the tyrosine residue. Molecular masses were determined by electrospray ionization mass spectrometry (ESI-MS) and compared against theoretical values. Peptide purity was assessed by RP-HPLC on a C18 column with an acetonitrile/water gradient containing 0.1% TFA, monitoring absorbance at 254 nm. Purity was expressed as the percentage area of the principal peak relative to total peak area. Conjugation of DEX to the peptide ε-amino group was confirmed by mass shift (ESI-MS) consistent with the expected product; MS/MS fragmentation analysis to directly confirm site-selective conjugation at the lysine ε-amine, as opposed to other potential nucleophilic sites, was not performed and is identified as a priority for future structural characterization.

### 4.5. Invert Vial Gelation Assay

The minimum gelation concentration (MGC) and qualitative gelation behavior of L-NapFFK(DEX)Y(p)-OH and D-NapFFK(DEX)Y(p)-OH were assessed by the invert vial method. Peptide–drug conjugates were weighed into glass vials and dissolved in PBS by the addition of 0.1 M NaOH with sonication (Grant XB ultrasonic bath), maintaining pH between 7.4 and 8.5 as monitored by a Hamilton MiniTrode pH sensor. Alkaline phosphatase (10 μL from the stock per vial, from a 1000 U/mL stock, diluted in PBS) was added to formulations at 2% *w*/*v*, mixed thoroughly, and incubated at 37 °C for 24 h. Gelation was assessed by inverting the vials immediately after ALP addition and at 24 h; absence of flow was taken as evidence of gel formation. The enzyme activities used throughout this study are supraphysiological and were selected to drive dephosphorylation rapidly and completely in every replicate, so that differences between the L and D enantiomeric systems could be attributed to the assembled network and the peptide–drug linkage rather than to differing extents of enzymatic conversion. They are not intended to model physiological gelation kinetics.

### 4.6. Oscillatory Rheology

The viscoelastic properties of L-NapFFK(DEX)Y(p)-OH at 2% *w*/*v* were characterized by oscillatory rheology on an Anton Paar MCR 302 rheometer (Graz, Austria) equipped with a cup-and-plate geometry. ALP (10 U) was added to 2 mL of freshly prepared precursor solution immediately before measurement, giving a final activity of approximately 5 U mL^−1^ (See Section 4.5). Time sweeps were conducted at 0.5% strain and 2.5 rad/s over 1200 min to monitor the sol–gel transition. Strain sweeps (0 to 1000% strain at 2.5 rad/s) were subsequently performed on the formed gel. The storage modulus (G′) and loss modulus (G″) were recorded throughout; G′ > G″ was taken as confirmation of hydrogel formation. Comparative rheology of the unconjugated parent sequences for L-NapFFKY(p)-OH and D- NapFFKY(p)-OH enantiomers was performed at 0.5% *w*/*v* under identical conditions of hydrogel formation mentioned in Section 4.5. Rheological characterization for the L-conjugate at 2% *w*/*v*; material availability precluded the equivalent measurement for the D-conjugate.

### 4.7. Transmission Electron Microscopy

The nanostructure of L-NapFFK(DEX)Y(p)-OH and D-NapFFK(DEX)Y(p)-OH hydrogels at 2% *w*/*v* was examined by transmission electron microscopy (TEM) at the Advanced Imaging Core Technology Unit, Queen’s University Belfast, using a JEOL JEM 1400 Plus microscope (JEOL UK Ltd., Hertfordshire, UK). Immediately after addition of ALP, 10 µL of the liquid precursor was deposited onto a carbon-coated copper TEM grid and incubated at 37 °C for 24 h to allow in situ gel formation. Grids were subsequently air-dried under a fume hood for 24 h before imaging at 40,000× magnification with a 200 nm scale bar.

### 4.8. In Vitro Drug Release

In vitro DEX release was evaluated for three formulations at 2% *w*/*v*: (i) L-NapFFK(DEX)Y(p)-OH physically mixed with an equivalent mass of free DEX, (ii) L-NapFFK(DEX)Y(p)-OH chemically conjugated, and (iii) D-NapFFK(DEX)Y(p)-OH chemically conjugated. Release was also evaluated for formulations (ii) and (iii) at 0.5% *w*/*v* to assess performance at the sub-MGC concentration. Hydrogels were formed in situ by adding ALP (10 U) to total volume of 2 mL of hydrogel formulation, and it was further divided into 100 µL aliquots of precursor solution in glass vials, followed by incubation at 37 °C for 24 h, giving final ALP activity of 5U mL^−1^. Release medium (5 mL pre-warmed PBS, pH 7.4) was gently layered above the gel without disruption. Vials were incubated at 37 °C with orbital agitation at 40 rpm. At predetermined timepoints over 28 days, 1 mL of release medium was withdrawn and replaced with fresh PBS to maintain sink conditions. DEX content was quantified by RP-HPLC (basis for cumulative % calculation described in Section 4.15). All experiments were performed in triplicate (*n* = 3).

### 4.9. Proteinase K Degradation Kinetics

The proteolytic stability of L-NapFFKY(p)-OH, D-NapFFKY(p)-OH, L-NapFFK(DEX)Y(p)-OH, and D-NapFFK(DEX)Y(p)-OH was assessed in the presence of proteinase K (PK). Each sequence was dissolved in PBS (pH 7.4) at 0.02% *w*/*v*. Aliquots of 1 mL were distributed into Eppendorf tubes, and PK was added at time zero to a final concentration of approximately 3 U/mL. Samples were incubated at 37 °C in a non-shaking incubator. At predetermined timepoints (0, 0.5, 1, 3, 6, 24, 72, 168, 336, 504, and 672 h), 100 µL aliquots were withdrawn and enzymatic activity was immediately quenched by addition of 50 µL glacial acetic acid. The remaining intact peptide was quantified by RP-HPLC and expressed as a percentage of the initial peak area at t = 0. Control samples without PK were processed identically. All experiments were performed in triplicate (*n* = 3).

The proteolytic stability of L-NapFFKY(p)-OH, D-NapFFKY(p)-OH, L-NapFFK(DEX)Y(p)-OH, and D-NapFFK(DEX)Y(p)-OH was assessed in the presence of proteinase K (PK), a broad-spectrum protease widely used as a standard surrogate for preliminary peptide biostability screening. PK is not specific to the ocular proteolytic environment; results obtained here should be interpreted as an indication of general proteolytic resistance rather than a direct prediction of in vivo ocular stability, which is discussed further in Section 2.8.

### 4.10. In Vitro Drug Release Under Proteolytic Conditions

To assess the impact of proteolytic activity on DEX release, L-NapFFK(DEX)Y(p)-OH at 0.5% *w*/*v* was evaluated under conditions of enzymatic challenge. Hydrogels were formed as described in Section 4.8. Release medium was supplemented with PK at a final concentration of approximately 3 U/mL. Sampling and HPLC quantification were performed as described above over 28 days.

### 4.11. Injectability

The injectability of L-NapFFK(DEX)Y(p)-OH and D-NapFFK(DEX)Y(p)-OH at 2% *w*/*v* was evaluated using a TA-TX2 texture analyzer (Stable Micro Systems, Godalming, UK) in compression mode. A concentric cylindrical probe (diameter 40 mm, depth 150 mm) was used to apply force at a rate of 2 mm/s to the plunger of a syringe fitted with a 30-gauge needle, loaded with 100 µL of formulation. Three replicates were performed per formulation, each with a fresh syringe and needle. Maximum injection force (N) and the area under the force-time curve (N·s) were determined from the resulting profiles. Water was used as a control reference.

### 4.12. Ex Vivo Scleral Permeation and Tissue Retention

Ex vivo permeation of DEX from L-NapFFK(DEX)Y(p)-OH and D-NapFFK(DEX)Y(p)-OH hydrogels was evaluated using porcine conjunctival–scleral tissue mounted on a modified Franz diffusion cell. Porcine eyes were obtained fresh, and conjunctival–scleral tissue was excised and mounted with the conjunctival side facing the donor compartment and the scleral side facing the receptor compartment (average scleral thickness ~1000 µm). The receptor compartment was filled with 5 mL of pre-warmed PBS (pH 7.4) maintained at 37 °C under continuous stirring.

A volume of 100 µL of peptide formulation was injected into the subconjunctival space immediately after addition of ALP to enable in situ hydrogelation within the tissue. At predetermined timepoints (1, 2, 3, 6, 12, and 24 h), 200 µL samples were withdrawn from the receptor compartment and replaced with fresh PBS. After 24 h, tissues were removed, homogenized in 0.5 mL PBS using a TissueLyser LT (QIAGEN, Venlo, The Netherlands) and extracted with 1 mL acetonitrile. Following centrifugation and filtration, the supernatant was analyzed by RP-HPLC to quantify tissue-retained DEX. Cumulative permeation and tissue retention data are reported as mean ± standard deviation (*n* = 3).

### 4.13. In Vitro Cytotoxicity

The cytotoxicity of Both peptide sequences conjugated with Drug (L-DEX and D-DEX) was assessed in ARPE-19 cells in accordance with ISO 10993-5:2009 guidelines. Cells were cultured in DMEM/F-12 supplemented with 10% heat-inactivated FBS and 1% penicillin-streptomycin at 37 °C in a humidified 5% CO_2_ atmosphere and seeded into 96-well plates at 2 x 10^5^ cells per well for 36 h prior to exposure. For indirect testing, each peptide formulation was incubated in serum-free DMEM/F-12 for 0 h, 24 h, 48 h, 72 h and 1 week to generate leachate extracts reflecting short-, medium-, and extended-term exposure; the resulting extracts were applied to cells for 24 h. Viability was expressed as a percentage relative to untreated control wells. All experiments were performed in triplicate (*n* = 3). Cell viability by resazurin reduction was used as the primary endpoint; more sensitive markers of sublethal toxicity (e.g., apoptosis, inflammatory cytokine release) were not assessed and are identified as priorities for future biocompatibility studies.

### 4.14. RP-HPLC Analytical Method for Peptide Purity and DEX Quantification

All RP-HPLC analyses were performed on an Agilent 1200 series system (Agilent Technologies, Santa Clara, CA, USA) equipped with a quaternary pump (G1311C), diode array detector, and autosampler, using a reverse-phase C18 analytical column. Chromatographic separation was achieved using a binary gradient of solvent A (0.1% *v*/*v* TFA in ultrapure water) and solvent B (0.1% *v*/*v* TFA in acetonitrile) at a flow rate of 1.0 mL/min over a 30 min run time, with UV detection at 254 nm. The initial mobile phase composition was 90% A/10% B, ramping to 10% A/90% B over 20 min, held for 5 min, then returning to initial conditions for re-equilibration. All injections were 10 µL. For peptide purity, each sequence was dissolved in DMSO/water (1:1 *v*/*v*) at approximately 1 mg/mL. Purity was calculated as the percentage area of the principal peptide peak relative to the total integrated peak area.

### 4.15. DEX Quantification and Calibration

DEX concentration in release, permeation, and tissue extraction samples was determined by RP-HPLC using the method described in Section 4.14. A stock solution of DEX (1 mg/mL) was prepared in acetonitrile and serially diluted in PBS to construct a calibration curve across the range of 0.1 to 100 µg/mL. Linearity was confirmed by linear regression (r^2^ > 0.999). The limit of detection (LOD) and limit of quantification (LOQ) were determined using signal-to-noise ratios of 3:1 and 10:1, respectively. For tissue retention samples, DEX was extracted from homogenized scleral tissue in acetonitrile (1 mL), centrifuged at 10,000× *g* for 10 min, and the supernatant filtered through a 0.22 µm PVDF membrane prior to injection. All sample concentrations were back-calculated from the calibration curve. Cumulative DEX permeated or released was expressed as a percentage of the total theoretical drug content per formulation.

### 4.16. Statistical Analysis

Statistical analyses were conducted using GraphPad Prism 9 (version 9.2.0). Where three or more groups were compared (in vitro drug release at 2% *w*/*v*, injection force, and cell viability), a one-way analysis of variance (ANOVA) with Tukey’s post hoc test was employed. Pairwise comparisons between two groups (in vitro drug release at 0.5% *w*/*v*, proteolytic stability, ex vivo scleral permeation, and tissue retention) were assessed using an unpaired Student’s *t*-test. A *p*-value of less than 0.05 was considered statistically significant. All results are expressed as mean ± standard deviation and were generated in triplicate (*n* = 3) unless otherwise stated.

## Figures and Tables

**Table 1:** ESI-MS Data and purity confirmation for the synthesized peptides and conjugates.

Compound	Expected *m*/*z* [M+H]+	Observed *m*/*z* [M+H]+	Mass Diff. (Da)	HPLC Purity (%)
NHS-DEX	590.2323	591.2405	1.008	N/A
L-NapFFK(DEX)Y(p)-OH	1325.5427	1326.5427	1.000	87.3%
D-NapFFK(DEX)Y(p)-OH	1325.5427	1326.5427	1.000	89.8%

## Data Availability

The raw data supporting the conclusions of this article will be made available by the authors on request.
